# Supplementary material for: Sensorimotor cortex beta oscillations reflect motor skill learning ability after stroke
Source: Brain Commun. 2020 Oct 7;2(2):fcaa161. doi: 10.1093/braincomms/fcaa161 (PMC7660041; doi:10.1093/braincomms/fcaa161)
Supplement: fcaa161_Supplementary_Data [file fcaa161_supplementary_data.docx]

**Sensorimotor cortex beta oscillations reflect motor skill learning ability after stroke**

Svenja Espenhahn, Holly E Rossiter, Bernadette CM van Wijk, Nellie Redman, Jane M Rondina, Joern Diedrichsen, Nick S Ward

**Supplementary Material**

**Methods**

### Clinical assessment

Stroke-related impairment was evaluated using the following standardized measures: the Action Research Arm Test (ARAT; 0–57) (Yozbatiran *et al.*, 2008), the Nine-Hole-Peg Test (NHPT; time to place 9 pegs into 9 holes) (Mathiowetz *et al.*, 1985*b*) and grip strength using a dynamometer (Mathiowetz *et al.*, 1985*a*). All scores were normalized to values of the unaffected arm of each patient. Since sensory loss is common after stroke (Tyson *et al.*, 2008), patients’ tactile sensitivity was tested using the Fugl-Meyer (FM) (Fugl-Meyer *et al.*, 1975) sensation and passive joint motion scale (0–12). In order to create a compound impairment score unaffected by floor and ceiling effects in individual scores (Ward *et al.*, 2003; Rossiter *et al.*, 2014), a principle component analysis (PCA) was performed on all measures (a lower PCA score corresponds to greater impairment).

Cognitive functioning was assessed using the Sustained Attention to Response Test (SART; max error score 225). To control for the effect of fatigue and sleep, patients completed computerised versions of the Fatigue Severity Scale 7 (FSS-7; 0–7) (Krupp *et al.*, 1989; Johansson *et al.*, 2014), the Neurological Fatigue Index (NFI; 0–7) (Mills *et al.*, 2012)) and the St. Mary’s Hospital sleep questionnaire (adapted from (Ellis *et al.*, 1981)) for the nights preceding testing. In order to generate a fatigue severity score, a PCA was performed on FSS and NFI ratings (lower PCA scores reflect lower levels of fatigue).

**Supplementary Table 1:** Stroke patient characteristics.

| **Age** | **Sex** | **Months since stroke** | **Lesion side** | **Affected hand** | **Suspected stroke etiology** | **ARAT** | **NHPT** | **Grip strength** | **Tactile sensitivity** | **FSS-7** | **NFI** |
| --- | --- | --- | --- | --- | --- | --- | --- | --- | --- | --- | --- |
| 74 | F | 136 | R | ND (L) | LACI | 100 | 91 | 97 | 12 | 3.7 | 2.2 |
| 71 | M | 41 | R | ND (L) | LACI | 100 | 77 | 92 | 12 | 2.8 | 1.5 |
| 57 | M | 80 | R | ND (L) | anterior thalamus | 100 | 97 | 93 | 12 | 1.6 | 1.8 |
| 50 | M | 43 | L | D (R) | posterior MCA | 98 | 68 | 89 | 12 | 6.1 | 2.5 |
| 63 | M | 122 | L | D (R) | striatocapsular | 100 | 91 | 90 | 12 | 3.6 | 1.8 |
| 63 | M | 70 | L | D (R) | LACI | 100 | 102 | 116 | 11 | 5.0 | 3.0 |
| 63 | F | 44 | R | ND (L) | frontal lobe | 100 | 95 | 67 | 12 | -3.1 | 2.9 |
| 71 | M | 220 | R | ND (L) | LACI | 96 | 50 | 67 | 11 | 4.0 | 2.5 |
| 56 | M | 49 | R | ND (L) | thalamus | 100 | 95 | 76 | 10 | 2.9 | 2.2 |
| 63 | F | 71 | L | AD (L) | LACI | 100 | 96 | 55 | 12 | 2.7 | 2.5 |
| 60 | M | 42 | L | D (R) | anterior MCA | 100 | 86 | 104 | 11 | 2.6 | 2.8 |
| 73 | M | 128 | L | D (R) | LACI | 100 | 89 | 87 | 12 | 3.9 | 2.4 |
| 71 | F | 57 | R | ND (L) | LACI | 100 | 124 | 39 | 12 | 4.4 | 2.6 |
| 75 | F | 136 | L | D (R) | PCA | 100 | 94 | 54 | 12 | 3.9 | 2.0 |
| 56 | M | 83 | R | ND L) | hypothalamus | 100 | 89 | 57 | 12 | 3.7 | 3.0 |
| 58 | F | 105 | L | D (R) | anterior MCA | 100 | 107 | 24 | 10 | 4.4 | 3.0 |

*M: Male; F: Female; R: Right; L: Left; ND: Non-dominant; D: Dominant; AD: Ambidextrous; LACI: Lacunar infarct; MCA: Middle cerebral artery; PCA: Posterior cerebral artery; ARAT: Action Research Arm Test; NHPT: Nine Hole Peg Test in peg/s; FSS-7: Fatigue Severity Scale; NFI: Neurological Fatigue Index*

*Individual target velocity*

Prior to the training, the average velocity with which the target moved along the arc was individually determined in order to ensure that the task was of equal difficulty for everyone at the beginning of the training and left enough room for improvement in performance. For this purpose, we implemented an adaptive staircase procedure, which, on any given trial, adjusted (increased/decreased) the target velocity dependent on the subject’s preceding performance until a pre-specified criterion range was reached. On average, patients reached the criterion in 15.5±5.1 trials and the number of trials required was comparable to the control subjects (*t_(1,34)_*=0.96, *p*=0.082). The individually determined target velocity with which subjects were subsequently trained on the continuous tracking task was applied to all sessions. Of note, the target velocity with which patients were trained (mean velocity ±SD = 45.38±5.22 deg/s) was significantly slower compared to the control group (mean velocity ±SD = 51.28±9.43 deg/s) [*t*_(34)_=-2.24, *p*=0.032].


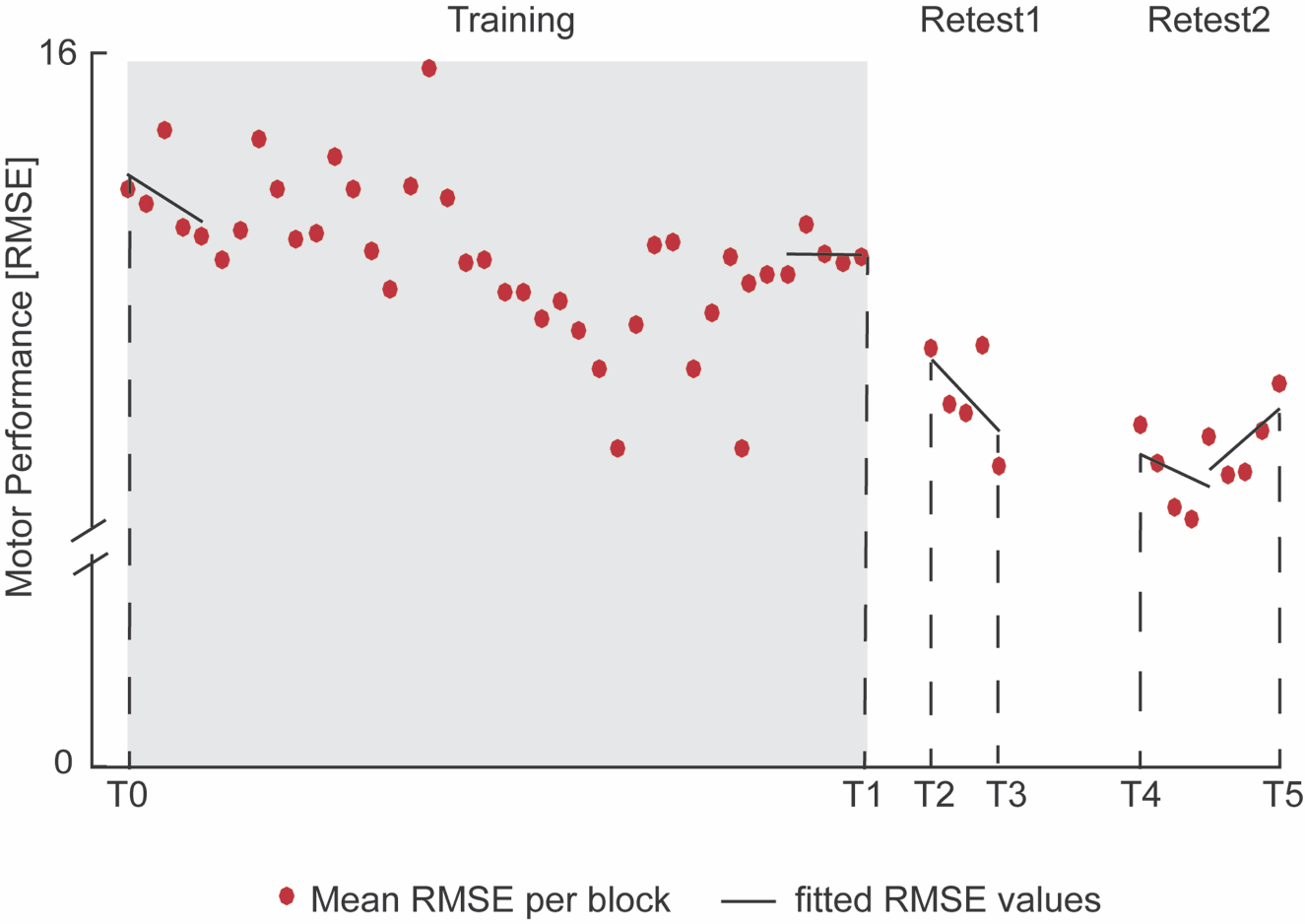


**Supplementary Figure 1:** **Linear regression approach for exemplary patient.** Dots represent individual blocks of an example patient during training and retest sessions of repeated sequence only. Black lines represent linear regression models across 5 blocks at the beginning and end of individual sessions. Corrected performance estimates were derived from these linear regression models at six different time points (T0 = first block of training, T1 = last block of training, T2 = first block of retest1, T3 = last block of retest1, T4 = first block of retest2, and T5 = last block of retest2) and used to subsequently assess changes in performance with training.

**Supplementary Table 2**: Potential predictors of performance at T2 and T4 included in the regression approach with LOOCV.

|  | **Performance at T2** | **Performance at T4** |
| --- | --- | --- |
| **Pre-training beta power measures** |  |  |
| Pre-BBc |  |  |
| Pre-BBi |  |  |
| Pre-MRBDc |  |  |
| Pre-MRBDi |  |  |
| Pre-PMBRc |  |  |
| Pre-PMBRi |  |  |
| **Post-training beta power measures** |  |  |
| Post1-BBc |  |  |
| Post1-BBi |  |  |
| Post1-MRBDc |  |  |
| Post1-MRBDi |  |  |
| Post1-PMBRc |  |  |
| Post1-PMBRi |  |  |
| Post2-BBc |  |  |
| Post2-BBi |  |  |
| Post2-MRBDc |  |  |
| Post2-MRBDi |  |  |
| Post2-PMBRc |  |  |
| Post2-PMBRi |  |  |
| **Motor performance at** |  |  |
| T0 |  |  |
| T1 |  |  |
| T2 |  |  |
| T3 |  |  |
| **Demographic information** |  |  |
| Age |  |  |
| Motor function |  |  |
| Cognitive function |  |  |
| Sleep characteristics |  |  |

**Supplementary Table 3**: ANOVA results of patients’ motor performance at different time points during the motor learning process relative to controls.

|  | Group | Time | Sequence Type | Interactions |
| --- | --- | --- | --- | --- |
| **Performance changes across initial training** | | | | |
| T0 vs T1 | *F_(1,33)_=0.01, p=0.330* | ***F_(1,34)_=9.69, p=0.004, ƞ_p_^2^=0.222*** | ***F_(1,34)_=15.73, p<0.001, ƞ_p_^2^=0.316*** | **time x group:**  ***F_(1,34)_=6.70,***  ***P=0.014,***  ***ƞ_p_^2^=0.165***  **time x sequence:**  ***F_(1,34)_=16.74,***  ***p<0.001,***  ***ƞ_p_^2^=0.330*** |
| **Performance changes after time delay (retest1, retest2)** | | | | |
| T1 vs T2 | ***F_(1,34)_=5.84, p=0.021,*** ***ƞ_p_^2^=0.147*** | ***F_(1,34)_=20.96, p<0.001, ƞ_p_^2^=0.381*** | ***F_(1,34)_=48.79, p<0.001, ƞ_p_^2^=0.589*** | **sequence x group:**  ***F_(1,34)_=4.39,***  ***P=0.044,***  ***ƞ_p_^2^=0.114*** |
| T3 vs T4 | ***F_(1,34)_=6.84, p=0.013,*** ***ƞ_p_^2^=0.167*** | ***F_(1,34)_=8.41, p=0.006, ƞ_p_^2^=0.198*** | ***F_(1,34)_=44.83, p<0.001, ƞ_p_^2^=0.569*** | **sequence x group:**  ***F_(1,34)_=5.56,***  ***p=0.024,***  ***ƞ_p_^2^=0.140***  **time x sequence:**  ***F_(1,34)_=9.07,***  ***p=0.005,***  ***ƞ_p_^2^=0.211*** |
| **Overall performance changes** | | | | |
| T0 vs T2 | *F_(1,34)_=1.03, p=0.317* | ***F_(1,34)_=50.39, p<0.001, ƞ_p_^2^=0.597*** | ***F_(1,34)_=20.49, p<0.001, ƞ_p_^2^=0.376*** | **time x group:**  ***F_(1,34)_=9.61,***  ***p=0.004,***  ***ƞ_p_^2^=0.220***  ***time x sequence:***  ***F_(1,34)_=29.53,***  ***P<0.001,***  ***ƞ_p_^2^=0.465*** |
| T0 vs T4 | *F_(1,34)_=1.30, p=0.262* | ***F_(1,34)_=56.25, p<0.001, ƞ_p_^2^=0.623*** | ***F_(1,34)_=6.99, p=0.012, ƞ_p_^2^=0.171*** | **time x group:**  ***F_(1,34)_=10.33,***  ***p=0.003,***  ***ƞ_p_^2^=0.233***  ***time x sequence:***  ***F_(1,34)_=12.74,***  ***P=0.001,***  ***ƞ_p_^2^=0.273*** |
| *Significant effects are indicated in bold. T0: beginning of training session; T1: end of training session; T2: beginning of retest1; T3: end of retest1; T4: beginning of retest2.* | | | | |

**Supplementary Table 4**: ANOVA results for spectral power measures

|  | Group | Hemisphere | Session | Interactions |
| --- | --- | --- | --- | --- |
| BB | *F_(1,34)_=0.21, p=0.653* | *F_(1,34)_=1.80,*  *p=0.188* | ***F_(2,68)_=5.90,***  ***p=0.004, n_p_^2^=0.148*** | n.s. |
| MRBD | *F_(1,34)_=2.22, p=0.146* | ***F_(1,34)_=21.06, p<0.001, ƞ_p_^2^=0.383*** | ***F_(2,68)_=5.94,***  ***p=0.004, ƞ_p_^2^*=0.149** | *n.s.* |
| PMBR | *F_(1,34)_=0.31, p=0.576* | ***F_(1,34)_=7.25, p=0.011, ƞ_p_^2^=0.176*** | ***F_(2,68)_=3.29,***  ***p=0.043, ƞ_p_^2^*=0.088** | n.s. |
| *Significant effects are indicated in bold. BB: Pre-movement baseline beta; MRBD: Movement-Related Beta Desynchronization; PMBR: Post-Movement Beta Rebound; n.s.: not significant.* | | | | |

**References**

Ellis BW, Johns MW, Lancaster R, Raptopoulos P, Angelopoulos N, Priest RG. The St . Mary ’ s Hospital Sleep Questionnaire : A Study of Reliability. Sleep 1981; 4: 93–97.

Fugl-Meyer AR, Jaasko L, Leyman I, Olsson S, Steglind S. The post-stroke hemiplegic patient. Scand J Rehab Med 1975; 7: 13–31.

Johansson S, Kottorp A, Lee KA, Gay CL, Lerdal A. Can the Fatigue Severity Scale 7-item version be used across different patient populations as a generic fatigue measure--a comparative study using a Rasch model approach. Health Qual Life Outcomes 2014; 12: 24.

Krupp L, LaRocca N, Muir-Nash J. The fatigue severity scale: application to patients with multiple sclerosis and systemic lupus erythematosus. Arch Neurol 1989; 46: 1121–1123.

Mathiowetz V, Kashman N, Volland G, Weber K, Dowe M, Rogers S. Grip and pinch strength: normative data for adults. Arch Phys Med Rehabil 1985

Mathiowetz V, Weber K, Kashman N, Volland G. Adult norms for the Nine Hole Peg Test of finger dexterity. Occup Ther J Res 1985; 5: 24–38.

Mills RJ, Pallant JF, Koufali M, Sharma A, Day S, Tennant A, et al. Validation of the Neurological Fatigue Index for stroke (NFI-Stroke). Health Qual Life Outcomes 2012; 10: 51.

Rossiter HE, Boudrias M-H, Ward NS. Do movement-related beta oscillations change after stroke? J Neurophysiol 2014; 112: 2053–2058.

Tyson SF, Hanley M, Chillala J, Selley AB, Tallis RC. Sensory loss in hospital-admitted people with stroke: characteristics, associated factors, and relationship with function. Neurorehabil Neural Repair 2008; 22: 166–72.

Ward NS, Brown MM, Thompson a J, Frackowiak RSJ. Neural correlates of motor recovery after stroke: a longitudinal fMRI study. Brain 2003; 126: 2476–2496.

Yozbatiran N, Der-Yeghiaian L, Cramer SC. A Standardised Approach to Perfroming the Action Research Arm Test. Neurorehabilitation & Neural Repair 2008; 22: 78–90.
